# An interview with Eustáquio A. Araujo

**DOI:** 10.1590/2177-6709.21.2.028-038.int

**Published:** 2016

**Authors:** James Leonard Vaden, Carlos Alberto Estevanell Tavares, Rolf Gordon Behrents, Orlando Tanaka

**Affiliations:** » Emeritus Professor, University of Tennessee, Department of Orthodontics, Health Science Center, Knoxville, TN, USA. » Private Practice, Cookeville, TN, USA » Former Clinical Associated Professor, University of Michigan, Ann Arbor, Michigan, USA. » Former Chairman and current Director of the American Board of Orthodontists. » Associate Director of the Tweed Study Course, Tucson, Arizona, USA. » Treasurer of the Tweed Foundation. » Editor of the Tweed Loop Journal. » DDS and Master in Orthodontics, University of Tennessee, Knoxville, TN, USA. » Graduated in History, Vanderbilt University, Nashville, TN, USA.; » President-Elect of the Brazilian Board of Orthodontics and Facial Orthopedics (BBO). » Fellow of the American Association of Orthodontists (AAO). » Fellow of the World Federation of Orthodontists (WFO). » Fellow of the Associação Brasileira de Ortodontia (ABOR). » Fellow of the Associação Gaúcha de Ortodontia (SOGAOR). » Fellow of the Associação Brasileira de Odontologia (ABO/RS). » Masters and PhD in Orthodontics, Universidade Federal do Rio de Janeiro (UFRJ), Rio de Janeiro, Rio de Janeiro, Brazil. » DDS, Universidade Federal do Rio Grande do Sul (UFRGS), Porto Alegre, Rio Grande do Sul, Brazil.; » Orthodontic Program Director of the Center for Advanced Dental Education, Saint Louis University, St Louis, MO, USA. » Lysle E. Johnston Jr. Professor of Orthodontics, Saint Louis University, Center for Advanced Dental Education (CADE), St Louis, MO, USA. » Editor-in-chief of the American Journal of Orthodontics and Dentofacial Orthopedics -AJO-DO. » Former Chairman of the Department of Orthodontics at Baylor College of Dentistry. Awarded an honorary doctoral degree by the University of Athens in Greece. » Past Faculty and Director of the Orthodontic Program, Case Western Reserve University, Cleveland, OH, USA. » Former Faculty member and Professor and Chairman of the Department of Orthodontics, University of Tennessee, College of Dentistry, Knoxville, TN, USA. » Author of "Growth of the Aging Craniofacial Skeleton." » Research associate of the Bolton-Brush Growth Study Center, Case Western Reserve University, Cleveland, OH, USA. » Won the prestigious Milo Hellman Award twice. » PhD from the University of Michigan, Ann Arbor, MI, USA. » Certificate and Master of Sciences from Case Western Reserve University, Cleveland, OH, USA. » DDS from Meharry Medical College, Nashville, TN, USA.; » Full professor, Pontifícia Universidade Católica do Paraná (PUC-PR), Undergraduate and Graduate Programs in Orthodontics, Curitiba, Paraná, Brazil. » Professor and Chairman of Orthodontics Department, Pontifícia Universidade Católica do Paraná (PUC-PR), Curitiba, Paraná, Brazil. » Post-doctoral fellowship at the Center for Advanced Dental Education at Saint Louis University, St Louis, MO, USA. » Diplomate of the Brazilian Board of Orthodontics and Facial Orthopedics. » Fellow of the American Association of Orthodontists (AAO). » Fellow of the World Federation of Orthodontists (WFO). » Fellow of the Associação Paranaense de Ortodontia (ABOR-PR). » PhD and Masters in Orthodontics, Universidade Federal do Rio de Janeiro (UFRJ), Rio de Janeiro, Rio de Janeiro, Brazil. » DDS, Universidade Federal do Paraná (UFPR), Curitiba, Paraná, Brazil.

**Figure:**
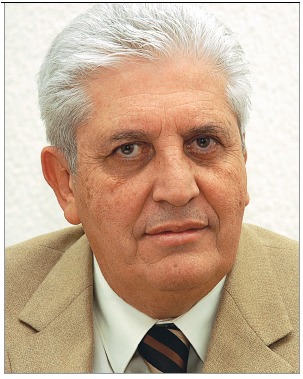

Professor Eustáquio Afonso Araújo received his DDS from Universidade Federal de Minas Gerais (UFMG), Belo Horizonte, Minas Gerais, Brazil, in the year of 1969. In 1981, he received his certificate in Orthodontics and Masters in Dental Sciences from the University of Pittsburgh, PA, USA. His professional life has been devoted to the orthodontic clinic, education and research. As he returned to Brazil in 1981, he initiated his academic career as an Assistant Professor at Pontifícia Universidade Católica de Minas Gerais (PUC-MG), a traditional and highly recognized institution in the country. He soon became involved with administrative duties as well, and was appointed Vice-director and later Director of the Biology and Health Sciences Institute. Later on he became Dean of the Dental School. In 2000, he applied for the Orthodontic Program Director position at Saint Louis University. He was selected and started his work at the end of that year; however, his commitment to PUC-MG in Brazil made him accept to return to his country in 2003 to lead a new vision for the institution. He then became President of PUC-MG with its 42,000+ students and 5,000 professors and employees. His presidency was marked by his dedication, for the new political vision and a strategic planning which led the institution to be voted and recognized as the best private university in Brazil. He left PUC-MG with 55,000+ students. During his tenure as PUC-MG president, Dr. Araujo never lost his ties with Orthodontics. He kept his private office in Brazil and worked with his associates in order to maintain himself close to his greatest passion: Orthodontics. During those four years, he continued to lecture at least two times a year at Saint Louis University. In 2007, his term as president of PUC-MG ended, and he did not accept a potential new term. He returned to Saint Louis University as a full-time professor and presently is the Associate Director for the Center of Advanced Dental Education, Orthodontic Clinic Director and the Pete Sotiropoulos Endowed Professor of Orthodontics. He has recently been awarded the Louis Jada Jarabak Award in recognition for his services to Orthodontics and academics. Dr. Araujo has given many contributions to orthodontic education through many researches, publications and lectures all over the world. Together with colleagues, he is responsible for a new textbook "Recognizing and correcting developing malocclusions." He is a member of the Brazilian Association of Orthodontics (ABOR), Angle Society of Orthodontics - Midwest Component, International College of Dentists, World Federation of Orthodontics, American College of Dentists, and is past President of the Brazilian Board of Orthodontics. His leadership contribution and high moral values have always been present in the many administrative and academic positions as well as strong community/political contributions. Dr. Araujo is married to Teresa Araujo, his soul mate and best friend, and has a daughter, Cristiana (Kika), an orthodontist and academician at the Jacksonville University in Florida, and a son, Francisco, a marketing major married to Veronica and who lives in Belo Horizonte. (Orlando Tanaka - interview coordinator )

O professor Eustáquio Afonso Araújo graduou-se em Odontologia pela Universidade Federal de Minas Gerais (UFMG), Belo Horizonte, Minas Gerais, Brasil, em 1969. Em 1981, recebeu o diploma de pós-graduação em Ortodontia e mestre em Ciências Odontológicas da *University of Pittsburgh*, PA, EUA. Sua vida profissional tem sido dedicada à prática clínica, formação e pesquisa em Ortodontia. De volta ao Brasil, em 1981, Eustáquio iniciou sua carreira acadêmica como Professor Assistente da Pontifícia Universidade Católica de Minas Gerais (PUC-MG), uma instituição tradicional e altamente reconhecida no Brasil. Em pouco tempo, ele assumiu funções administrativas e foi nomeado Vice-diretor e, depois, Diretor do Instituto de Ciências Biológicas e da Saúde na mesma universidade. Mais tarde, tornou-se Diretor da Faculdade de Odontologia. Em 2000, candidatou-se para a posição de Diretor do Programa em Ortodontia da *Saint Louis University*. Foi selecionado e começou a trabalhar no final daquele mesmo ano; porém, seu comprometimento com a PUC-MG no Brasil levou-o a retornar ao seu país em 2003, para contribuir com novas perspectivas para a instituição. Tornou-se, então, Reitor da PUC-MG, com seus mais de 42 mil alunos e 5.000 professores e funcionários. Seu mandato foi marcado por sua dedicação, visão política inovadora e um planejamento estratégico que levou a instituição a ser votada e reconhecida como a melhor universidade particular do Brasil. Prof. Eustáquio deixou a PUC-MG com mais de 55 mil alunos. Durante seu mandato enquanto Reitor da PUC-MG, Dr. Araujo jamais desfez seus laços com a Ortodontia. Ele manteve sua clínica particular no Brasil e trabalhou com seus sócios para manter-se próximo à sua grande paixão: a Ortodontia. Durante esses quatro anos, continuou a ministrar aulas duas vezes ao ano na *Saint Louis University*. Em 2007, o mandato como Reitor da PUC-MG terminou e ele não aceitou uma possível reeleição. Em vez disso, retornou à *Saint Louis University* como Livre-Docente e, atualmente, é Diretor Associado do *Center of Advanced Dental Education*, Diretor Clínico de Ortodontia e Professor Titular da Cadeira Pete Sotiropoulos de Ortodontia. Recentemente, recebeu o prêmio Louis Jada Jarabak, em reconhecimento pelos serviços prestados à Ortodontia e à academia. Foram muitas as contribuições do Dr. Araujo para a formação em Ortodontia, por meio de inúmeras pesquisas, publicações e aulas ministradas ao redor do mundo. Em parceria com alguns colegas, é responsável pelo livro "*Recognizing and correcting developing malocclusions*". É, ainda, membro das seguintes instituições: Associação Brasileira de Ortodontia (ABOR), *Angle Society of Orthodontics* - Midwest Component, *International College of Dentists*, Federação Mundial de Ortodontia e *American College of Dentists*, e é Ex-presidente do Board Brasileiro de Ortodontia e Ortopedia Facial (BBO). Sua contribuição de líder e seus valores morais sempre estiveram presentes em muitas das funções administrativas e acadêmicas que assumiu, além de suas contribuições à comunidade e à política. Dr. Araujo é casado com Teresa Araujo, sua alma gêmea e melhor amiga, com quem tem uma filha, Cristiana (Kika) - ortodontista e professora na *Jacksonville University*, Flórida -, e um filho, Francisco, publicitário, casado com Veronica, morando em Belo Horizonte. (Orlando Tanaka - coordenador da entrevista)

## How did you choose your path in life? Rolf G. Behrents

I believe I came to this world with the objective of working with people. My "craziness" for academics was natural. If I were not doing what I have chosen to do in Dentistry, I would probably be doing a similar work in another health profession. My life as the youngest of a family of sixteen was strongly marked by the presence of my parents. My mother was always the strength of the family; my father was a dentist since the 1930s, working in rural areas and taking his chair from farm to farm in order to provide for all of us. Throughout my life, I always mirrored the examples of my father: a hardworking man who dedicated his life to the family, sports and music. Since 1981, every single day, I attempt to live by his examples in the mentoring of our residents. It seems like my choice was natural, I could not see myself doing anything else. 

## What do you see as the greatest challenge to orthodontic education in the next five years? James L. Vaden

It is not possible to analyze orthodontic education without segregating it geographically and culturally. The scenario in North America is quite different from Asia, Europe and South America. Let me establish a parallel between the USA and Brazil. In the USA, the number of graduate courses is controlled by ADA (American Dental Association) and its austere accreditation. Presently, there might be 60-65 graduate programs in the whole country, which educates around 300-350 new orthodontists every year, a number that seems to be adequate for the replacement of those who retire. The biggest challenges here are more related to student debt and the "new" trend towards corporate practices, more frequently run by professionally trained CEOs who are not necessarily involved with Orthodontics. Unlike other countries, higher education in the USA is not free, it is paid and well paid. To become an orthodontist, a student must undergo four years of college education, four years of dental school and two to three years of orthodontic education. That adds up to 10 or 11 years of preparation and paid schooling. At a very low average price calculation, considering US$ 30,000/year (maybe a very low estimate), the investment comes to a grand total of US$ 300,000-400,000. On top of that, many students also request money for living expenses and, in addition, large percentages are married and have kids. Soon after graduation, they have to start paying back their student loans. First big challenge: how are they supposed to set up private practice or buy one out with such a huge financial responsibility? Second challenge: should they increase their debt or work for someone to start "making money" to pay the bills? When confronted with this dilemma, many recent graduates opt to join corporations that were able to foresee this situation and make it an opportunity for great "business." In my mind, however, the crucial question is even more complex: business or quality? Is it possible to maintain quality when you are expected to see around 100+ patients a day? 

In Brazil, there is an uncountable number of courses - I am sorry, but I cannot consider the vast majority of graduate programs - in addition to university programs that seriously dedicate to a stronger formation of specialists who eventually suffer the competition from the weekend, sometimes monthly, part-time "money makers." 

In Asia and Europe, as in the USA, the problem is not related to the number of specialty programs. The system requires 36 months of full-time preparation. The number of programs could probably be doubled. In many countries, Dentistry is socialized and the government provides much but not all. Additionally, the economies may not be strong enough to allow a majority of patients to seek private care. 

In conclusion, what I see in the USA and Canada is frequently an unmanageable student debt leading many of our residents to employment increasingly offered by multi-office corporations whose focus is mostly on production and, in many instances, is in conflict with quality of diagnosis, treatment and ethics. Outside North America, there are other types of pressures, many associated to ethics as well. 

## Both experienced orthodontists as well as recent graduates deal with complex cases. What is more common to see in the daily practice? Can you exemplify it? Orlando Tanaka

Open bite and Class III are situations of greater complexity, but other routine treatment problems may also occur. Instead of listing treatment modalities, I would rather list the most common problems we see in a large clinic like ours at Saint Louis University. I am not afraid to say that a frequent problem is uncontrolled anchorage. It is not rare to see severe anchorage loss due to little attention to mechanics. How to overcome it? Establish a Class I canine relationship as early as possible and do not make treatment more complex for not paying attention to details. Another routine problem is not following the evolution of treatment in a proper way. My advice is to take photographs at least every other appointment and check them on a regular basis at every visit. Nowadays, with the advantage of digital photographs and updated offices, it is much easier to keep track of your work at every visit. Evaluate mechanics monthly and make decisions based on careful evaluations. 

## What recent developments do you consider significant advancements in Orthodontics? Rolf G. Behrents

Anchorage control and CBCT. As mentioned before, the advent of bone anchorage has made the orthodontists' life much easier. Compliance has and had always been one of the major variables for successful results. In terms of 3D diagnostic tools, although further studies are still needed, I believe it has been a major step forward in diagnosis. In relation to impacted teeth/developmental deviations, I actually believe that in the past we were kind of guided by the hands of God. How many situations did we confront without a good and solid diagnosis in the past? 

## Orthodontic treatment, regardless of technique, should shoot for the most ideal results possible. Why is there so much controversy over some techniques? Can you mention a few examples? Orlando Tanaka

My answer starts with a simple statement: "Teeth are dumb and they do not know where they are going." An analogy that I frequently make is that if one knows how to drive, he/she can probably drive any car. Technique controversies are normally related to propaganda and "magic brackets." A good clinician should be able to treat with any type of bracket. With the advent of new wires, it is even easier to achieve treatment goals. Let's stress, especially to the young, that diagnosis is the key to a successful treatment. There is no magic in Orthodontics; let's try not to be overwhelmed by vendors. The core (heart) of Orthodontics is and will always be diagnosis. 

## In which cases would you consider skeletal anchorage as your first treatment option? Carlos Alberto Estevanell Tavares

I have very little, if any, experience with skeletal anchorage, I mean mini plates. I have actually used it a couple of times in Class III adolescent patients attempting non surgical treatment. I was not very successful, partially because even though it is a great technique, it is not 100% compliance-free. I needed patient cooperation and did not receive it. I have seen great results from other colleagues. It is important to stress that in order to be successful one must also rely on an experienced oral-surgeon familiar with the procedure. 

## TADs/mini screw anchorage devices have been used by the specialty for the past ten or so. Do you think that their true and useful role has now been defined? Is their use going to become increasingly popular or is it going to be limited to specific malocclusions that present with specific problems? James L. Vaden

Part of my answer is actually an "Amen" to those who like me put common sense ahead of technology trends. This is how I see it. Initially there was a craziness for TADs, partially because they were modern (you would be outdated if you were not using them), new and "*chic*", as the French would say it. In the beginning, a lot was done without a thorough knowledge of how and when to use them. With the number of disappointments and failures, and maybe because patients did not want extra costs, our colleagues, nowadays, start to view them as treatment facilitators. I, myself, am not a TAD maniac. Actually, one of the lectures in my portfolio, and which I love, is named "There is life without TADs." In my humble opinion, bone anchorage is certainly a great adjunct mostly indicated for adult orthodontic mechanics. As an academician, I deal with residents daily and they all show high interest in learning and getting involved with TADs. They are absolutely correct; this is the right time to learn. However, I like to reinforce the necessity to learn alternative ways to achieve the same goals without TADs. But, please, do not take me wrong. As mentioned before, I consider bone anchorage one of the greatest advances in Orthodontics, but common sense must be exercised in their use. It seems like the TADs mania is probably dissipating, but we must remember that those devices can be extremely useful when used properly. 

## How do you treat non skeletal open bite in adults? Carlos Alberto Estevanell Tavares

This is a complex question, but let me try to cover it as much as possible. Beginning with Norman Kingsley, the "grandfather of Orthodontics," in the 19^th^ century,^1^ anterior open bites have proven to be one of the most difficult malocclusions to treat and retain.^2^ ^,^ ^3^ There are many reasons for this, and many factors that need to be kept in mind when attempting to tackle the difficult task of correcting an open bite. Much of the difficulty lies in the fact that open bite etiology is often multi factorial and the relationships of teeth, soft tissues and skeletal structures are all important when diagnosing, treating, and maintaining correction of an open bite malocclusion.^4^

When the skeletal numbers are within normal range, my first step is to try to identify the reason for the open bite. If there is a habit or a strong function (tongue) component, I recommend tongue spurs as auxiliary. For adult patients, it may sound unreachable, but if the patient is really interested in a good and more stable result, I believe it is our responsibility at least to attempt to control one of the possible causes of the problem. 

In many situations, adult patients also present remarkable anxiety and want fast results. Should that be the case, a combination of orthodontics with maxillofacial surgery may be the best way to handle it. It is important to say that the etiology must be addressed even if surgery is the selected route. A lot depends on the facial type and the dental malocclusion. 

Addressing your question with a more generic answer, normally, if there are no skeletal vertical deviations, the procedures include one or two jaw surgeries, segmental procedures, corticotomies and/or extractions. When surgery is not an option, extractions become the solution. With the advent of better anchorage, mini-implants or miniplates, the possibility of success increases. 

The side effects of extractions in open bite patients are still debatable at this time. How does posterior intrusion affect the overall result? Does it provide long lasting results? What is the effect on gingiva display (gumminess)? Which extraction pattern is accompanied by more detrimental side effects? As we can see, there are still many questions to be answered. 

We, Dr. Daniel Floyd and I, have just finished a study "The Positions of Maxillary Incisors in Anterior Open Bite Cases" with the purpose of evaluating the vertical and horizontal position of maxillary incisors when comparing non extraction and extraction of maxillary first premolar orthodontic treatments in anterior open bite patients. The study also attempted to establish a correlation of incisors extrusion with gingival display. The results of the study show that maxillary central incisors extruded similar amounts in extraction and non extraction treatment of anterior open bite. The findings of this study do not support the rationale behind the common assumption that extracting permanent teeth unequivocally causes an increase in maxillary incisor extrusion and gingival display compared to non extraction treatment due to vertical extrusion of maxillary incisors alone. According to the data, changes relative to hard and soft tissues visible on the cephalograms can shed some light onto how maxillary gingiva would likely respond to treatmen, but no definite conclusion could be derived from the study.^5^

**Figure 1:**
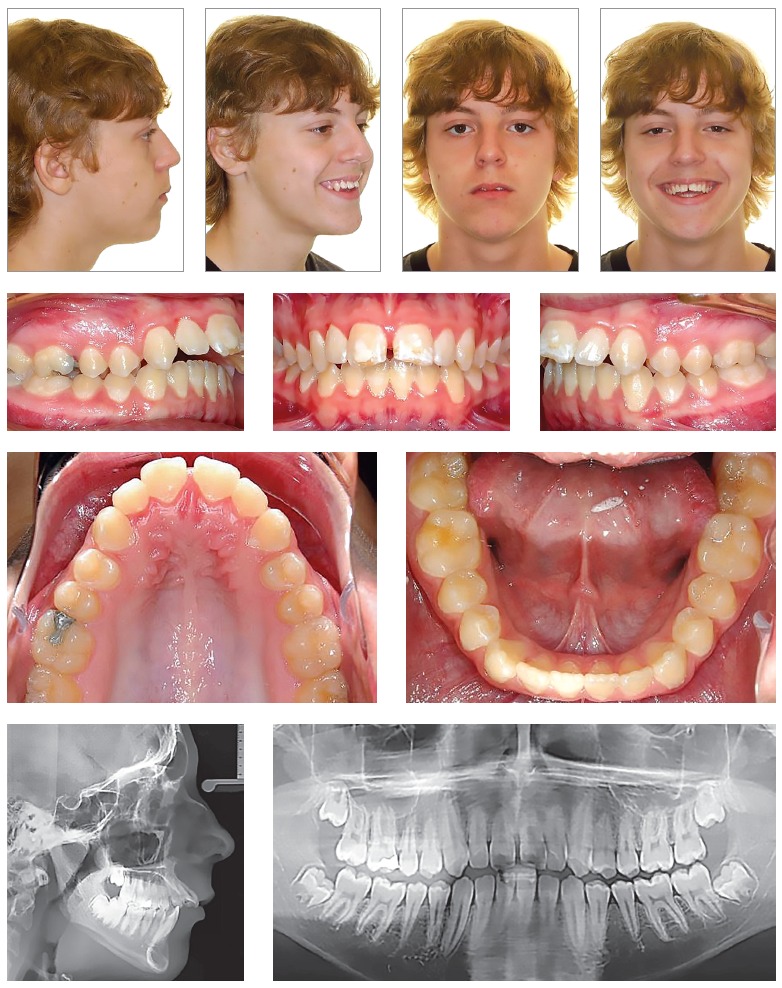
Pretreatment records of a 15-year-old male who presented for treatment with the major complaint of "sticking out teeth." Facial and intraoral photographs as well as radiographs show a severe Class II hyperdivergent malocclusion. History of bicycle accident and trauma of maxillary central incisors. Facial features indicate posterior gumminess. Due to the severity of the open bite, the large restoration on the maxillary right first molar, and the shortening of the maxillary central incisors roots, the proposed treatment plan was the extraction of first maxillary molars with anchorage control by means of palatal TADs and a TPA.

**Figure 2:**
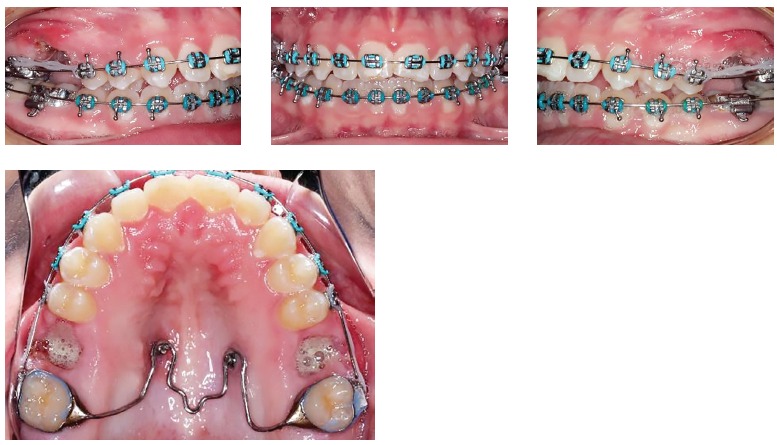
Progress. Extraction of both maxillary first molars and distalization of anterior teeth anchored on a TPA with mini-implants.

**Figure 3:**
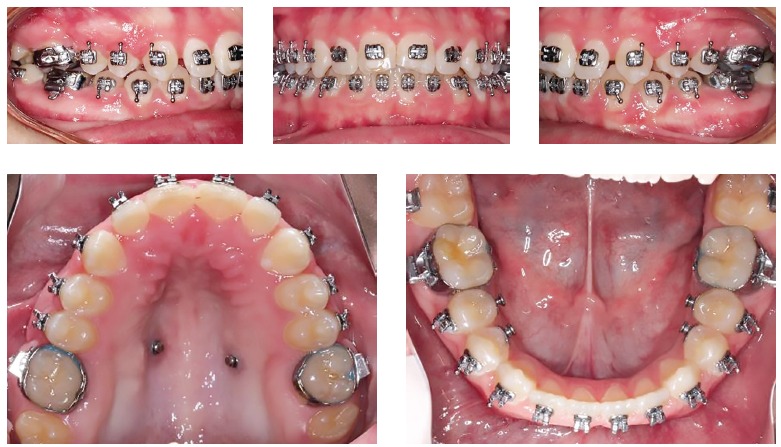
Progress. Extraction spaces closed, canine and molar in Class I relationship, and eruption of maxillary third molars.

**Figure 4:**
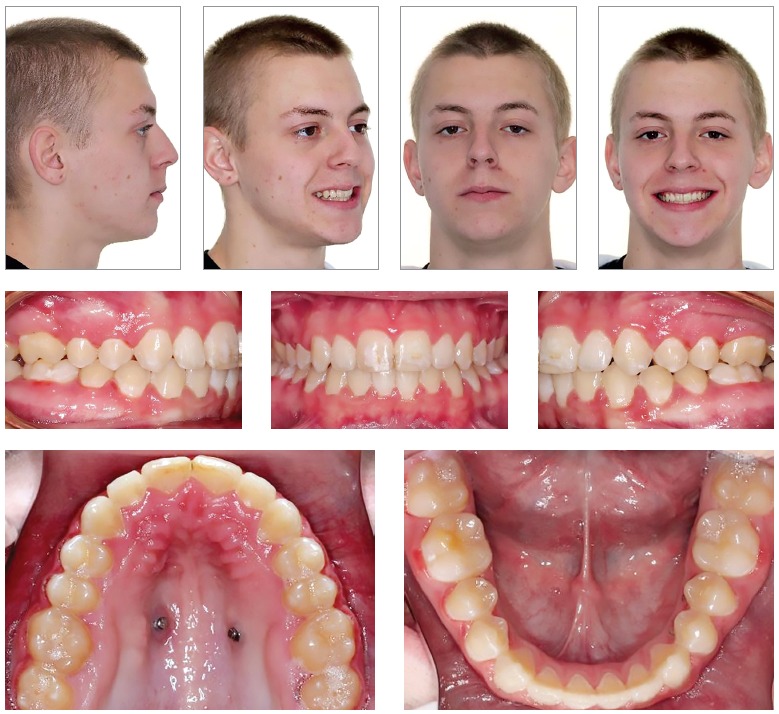
Post-treatment records at 17 years of age, after two years of orthodontic treatment.

**Figure 5:**
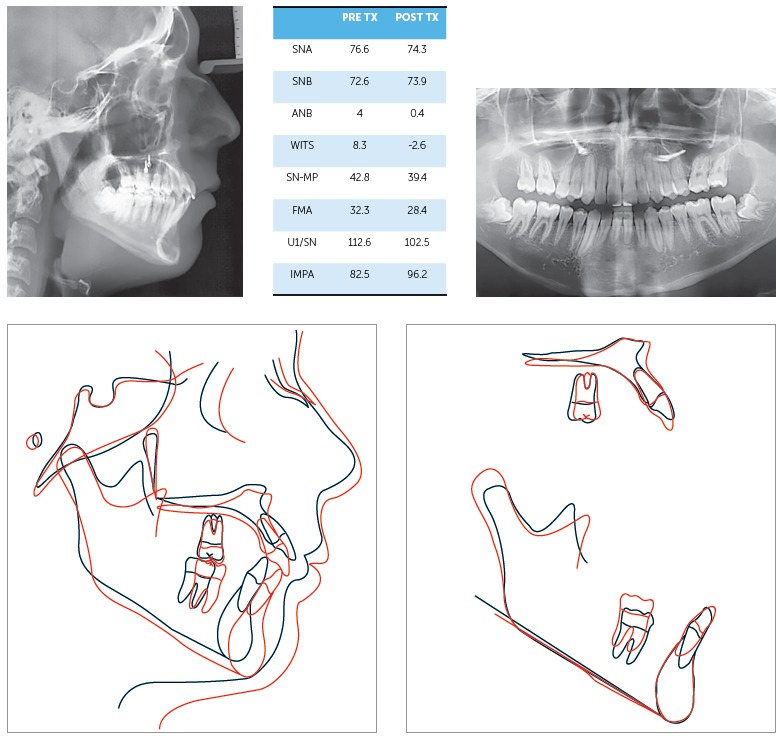
Cephalometric measurements table, and superimpositions: black line, pretreatment; Red line, post-treatment. Treatment objectives were achieved with good Class I relationship and excellent vertical control. The patient was less hyperdivergent at the end of treatment.

Among the extraction options, I would like to mention that I have been using the strategy of extracting maxillary first molars even though the literature does not present evidence that the so called wedge effect necessarily occurs. I have attached one of my results for a patient treated with maxillary first molar extractions (Figs 1-5). 

## In which situations would you have special concerns about retention? Carlos Alberto Estevanell Tavares

Retention is in fact one of the areas of biggest concerns in Orthodontics. What is stable? I could say that there is nothing more stable than the malocclusion. No matter what we do, it seems like the odds of more or less relapse is present. Some types of malocclusion, such as open bite, present so many variables that controlling post treatment changes are a battle. Another malocclusion of concern is Class III. If correction is done in adults, there is a reduced risk of relapse, but when adolescents with effective growth potential are involved, the need for a second intervention after growth ceases is immense. Nature does not help and patients must understand that as a human being gets older, so do the teeth. Aging may bring increasing grey hair as well as wrinkles on the face. Teeth are not different, the occlusion ages and permanent retention may reduce some of its impact on alignment and post orthodontic results. 

## How important will board certification be for the next generation of orthodontists? James L. Vaden

In my opinion, board certification is the Oscar in Orthodontics. It took me a long time to become certified. When I decided to pursue the boards, I did it twice, the Brazilian and the American Board of Orthodontics. The preparation for the clinical exam imposes an auto evaluation on you. You see great things you have done and also there is much *mea culpa* along the way. It is the best auto assessment on diagnosis, treatment planning, clinical skills, and office organization. I have no doubt I became a better orthodontist after I completed mine and I was also able to evidence my overall failures, especially on record keeping and quality assessment. 

Board certification is the standard to be followed towards the so longed-for excellence. It may be the best way to define commitment to excellence. As an academician and a person who also has lived the reality of Brazilian Orthodontics, I invite all to read a recent editorial I had the pleasure to write for Dental Press: "The Pursue of Quality^6^" in which I invited our colleagues to stand out from the crowd through board certification. It definitely differentiates professional quality. In Brazil, we have a great contrast, excellent and outstanding orthodontists and those who became the victims of an immoral system and were forced to surrender themselves to the "street corner market of orthodontic education", to the vendors of cheap training that has spread so rapidly. It undermines the country reputability and respect. We are living the braces craze, a fashion taken up with enthusiasm by opportunists. Board certification can definitely separate the wheat from the chaff. 

## If you could ask God one question about the art and science of Orthodontics (and God promises he will provide the answer, but only to one question), what question would you ask? Rolf G. Behrents

"Dear God, why do some want to destroy Orthodontics?"

My answer has to do with the many "new" approaches to provide treatment (it does not necessarily mean good treatment). I fear that the focus has been shifting away from diagnosis and I do feel sorry for the young professionals who, besides a fair competition, will have to fight poor treatment delivery, including over the counter gadgets. 

## As you have been away from Brazil, how do you see the future of Brazilian Orthodontics? Carlos Alberto Estevanell Tavares

On one hand, I see it with happiness because there are many serious educators in the country. On the other hand, I have reservations because of the commercial goals of orthodontic education. As leaders and role models, it is our responsibility to demonstrate work ethics, devotion and dedication. 

As mentioned on my editorial "The Pursue of Quality,^6^" it is scary when you see yourself in the shoes of our young colleagues - recent graduates or about to graduate in Dentistry - who normally are full of plans and dreams. New ideas pop up in their minds for a brilliant future. At that point, however, they are forced to face an unfair reality: the market. Then they are approached by unscrupulous "sellers of illusions" with false promises to make them orthodontists within a very short amount of time, with little commitment, and the miracle of having the doors of happiness open for them. Pure illusion! Pure fantasy! This type of cheating is a more generalized standard than some may think. 

## We understand that you and Dr. Buschang have just finished the edition of a textbook. What is it about and what will it add to the present literature? Orlando Tanaka 

We are actually very excited about our work and how it may contribute to the specialty. We named it "Recognizing and correcting developing malocclusions, A problem-oriented approach to Orthodontics."^7^ As you can see from the title, we do expect to follow a child's development, recognizing deviations from normal and presenting solutions. It presents a very good review in growth and development, but it is done in a different format. We approach growth and/or development in relation to each malocclusion, detect problems and address them. How does a Class I grow and what could go wrong? How does a Class II grow and what could go wrong? How does a Class III grow and what could go wrong? 

Besides this piece of information, there are contributions on genetics, missing teeth, eruption deviations, habits, autotransplantation and biomechanics. 

We are excited with the final product. We believe it is a great contribution to Orthodontics and Pediatric Dentistry and also a reference for graduate students.
